# Adult-Onset Xanthogranulomatous Inflammation Presenting as a Cervical Branchial-Cleft Cyst

**DOI:** 10.7759/cureus.85215

**Published:** 2025-06-02

**Authors:** Daiki Onodera, Shinichi Oikawa, Yoshihiko Koike, Aya Katsura, Ryoukichi Ikeda

**Affiliations:** 1 Department of Otolaryngology, Head and Neck Surgery, Iwate Medical University, Yahaba, JPN; 2 Department of Otolaryngology, Head and Neck Surgery, Iwate Medical University School of Medicine, Yahaba, JPN

**Keywords:** branchial-cleft cyst, cervical cyst, frozen section, neck tumor, xanthogranulomatous inflammation

## Abstract

Xanthogranulomatous inflammation (XGI) is a rare benign histiocytic reaction in the head and neck. Adult-onset XGI within a branchial-cleft cyst (BCC) is exceptionally uncommon, with only two cases previously reported. We present the oldest documented patient and review the literature. A 71-year-old man presented with a painless 3-cm right lateral-neck mass. Ultrasound and MRI suggested a benign thin-walled cyst; fine-needle aspiration revealed foamy macrophages without atypia. Intraoperatively, dense fibrosis and firm adhesion to the carotid sheath raised concern for metastatic carcinoma. Frozen-section analysis demonstrated foamy histiocytes and Touton giant cells without epithelial atypia, confirming XGI. En-bloc excision was completed without radical neck dissection. Recovery was uneventful, and no recurrence was observed at 14 months. Only three adult BCC-XGI cases have been reported, including the present patient. All showed deceptively benign imaging, disproportionate intraoperative fibrosis, and reliance on frozen section to exclude malignancy. Chronic leakage or rupture of cyst contents - possibly potentiated by fine-needle aspiration - appears to trigger the xanthogranulomatous response. Surgeons should consider XGI when encountering fibrotic lateral cervical cysts in older adults. Prompt intraoperative frozen-section analysis enables conservative excision and prevents overtreatment.

## Introduction

Branchial cleft cysts (BCCs) are congenital epithelial remnants arising from incomplete obliteration of the cervical sinus during the 2nd-7th embryonic weeks [1,2]. Approximately 90 % originate from the second cleft, and 75 % are diagnosed between 20 and 40 years [1]. Although BCCs represent the commonest cystic neck masses in young adults, new lateral cystic lesions in patients > 40 years warrant comprehensive evaluation because metastatic cystic squamous‑cell carcinoma becomes more prevalent with age [3,4]. Xanthogranulomatous inflammation (XGI) is a benign histiocytic process characterized by foamy macrophages and Touton-type giant cells [5]. While XGI is well recognized in the kidney and gallbladder, head-and-neck involvement is rare [6,7]. Only two adult cases of BCC complicated by XGI have been reported [8,9]. We report a unique case of an elderly patient with a lateral cervical cyst complicated by XGI in which intraoperative differentiation from malignancy presented a significant diagnostic challenge.

## Case presentation

**Figure 1:**
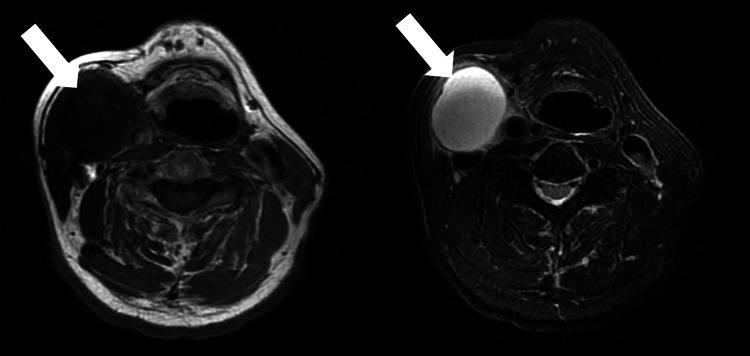
T1-weighted MRI (left) and T2-weighted MRI (right) A T1-hypointense/T2-hyperintense lesion (white arrow) was observed.

A 71-year-old man presented to our department with a painless, elastic 30-mm swelling deep to the right sternocleidomastoid muscle for 2 days. His past medical history included appendectomy and benign prostatic hyperplasia; he smoked 15 cigarettes per day for 48 years. The physical examination revealed no abnormal or pathological findings in the oral cavity, pharynx, or larynx. Ultrasonography demonstrated a 37 × 29 mm well-demarcated anechoic cyst with internal hyperechoic debris, and MRI revealed a thin-walled T1‑hypointense/T2‑hyperintense lesion abutting the carotid sheath with no mural nodules (Figure 1). It would be good to note that there were no findings suggestive of infiltration into the carotid sheath. Based on these imaging findings, the differential diagnosis included a neck abscess and lymphadenopathy on ultrasound, whereas the MRI characteristics raised suspicion for cystic lymph node metastasis.　

Fine‑needle aspiration (FNA) produced proteinaceous fluid rich in foamy macrophages but no atypical cells (class I), and the lesion was scheduled for excision under the working diagnosis of a BCC.

**Figure 2:**
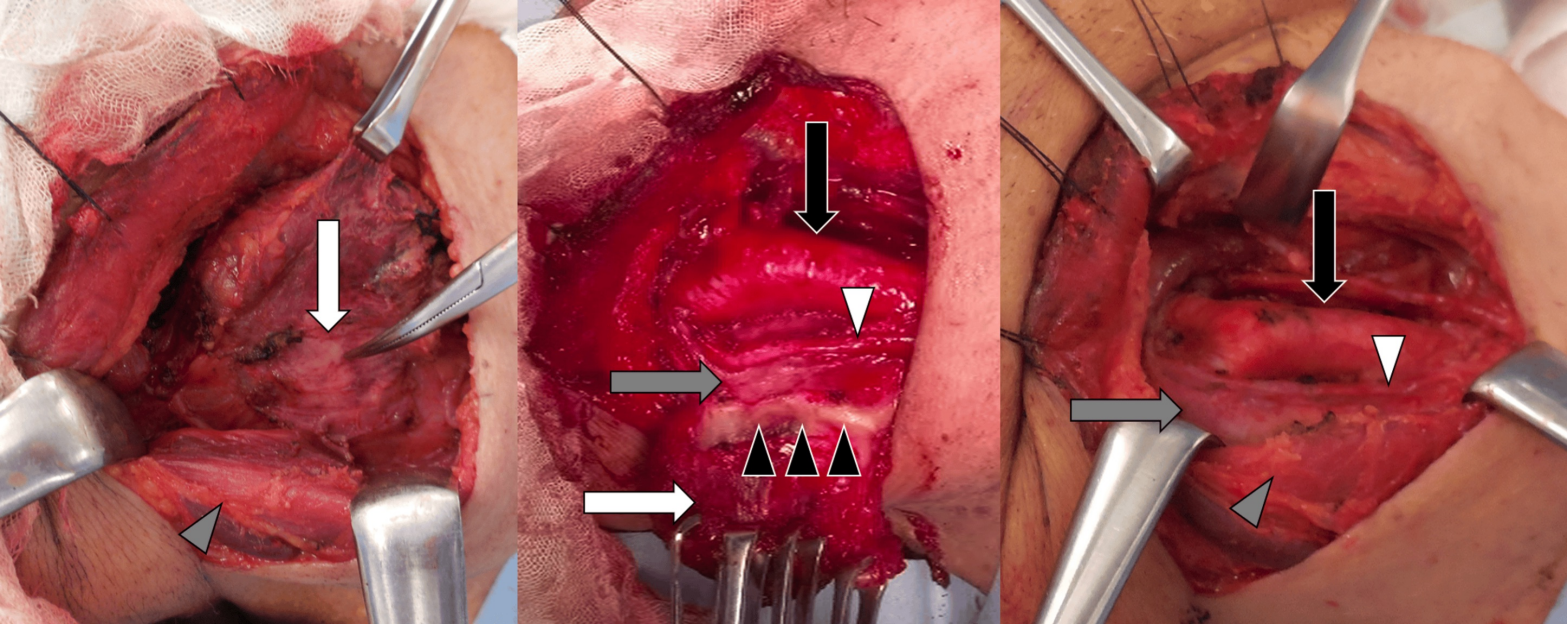
Intraoperative findings Left: The tumor (white arrow) was dissected free from the anterior border of the sternocleidomastoid muscle (gray arrowhead). Middle: The medial aspect of the tumor (white arrow) was densely adherent to the internal jugular vein (gray arrow) at the area of fibrosis (black arrowhead). The white arrowhead identifies the vagus nerve, and the black arrow denotes the common carotid artery. Right: Post-excision view. Gray arrowhead: sternocleidomastoid muscle; gray arrow: internal jugular vein; white arrowhead: vagus nerve; black arrowhead: common carotid artery.

Through a transverse cervical incision, subplatysmal flaps were raised, and the thinned sternocleidomastoid was reflected (Figure 2: left). The cyst was circumferentially fibrotic and firmly adherent to the carotid sheath (Figure 2: middle). Suspecting metastatic carcinoma, such as HPV-associated oropharyngeal cancer or papillary thyroid cancer, we submitted biopsies from both lateral and medial walls for the intraoperative frozen section. The lateral wall showed fibrosclerotic tissue, whereas the medial wall revealed foamy histiocytes and Touton giant cells without atypia, consistent with XGI. Guided by this benign diagnosis, the cyst was sharply dissected from the vascular sheath and removed en bloc (Figure 2: right).

**Figure 3:**
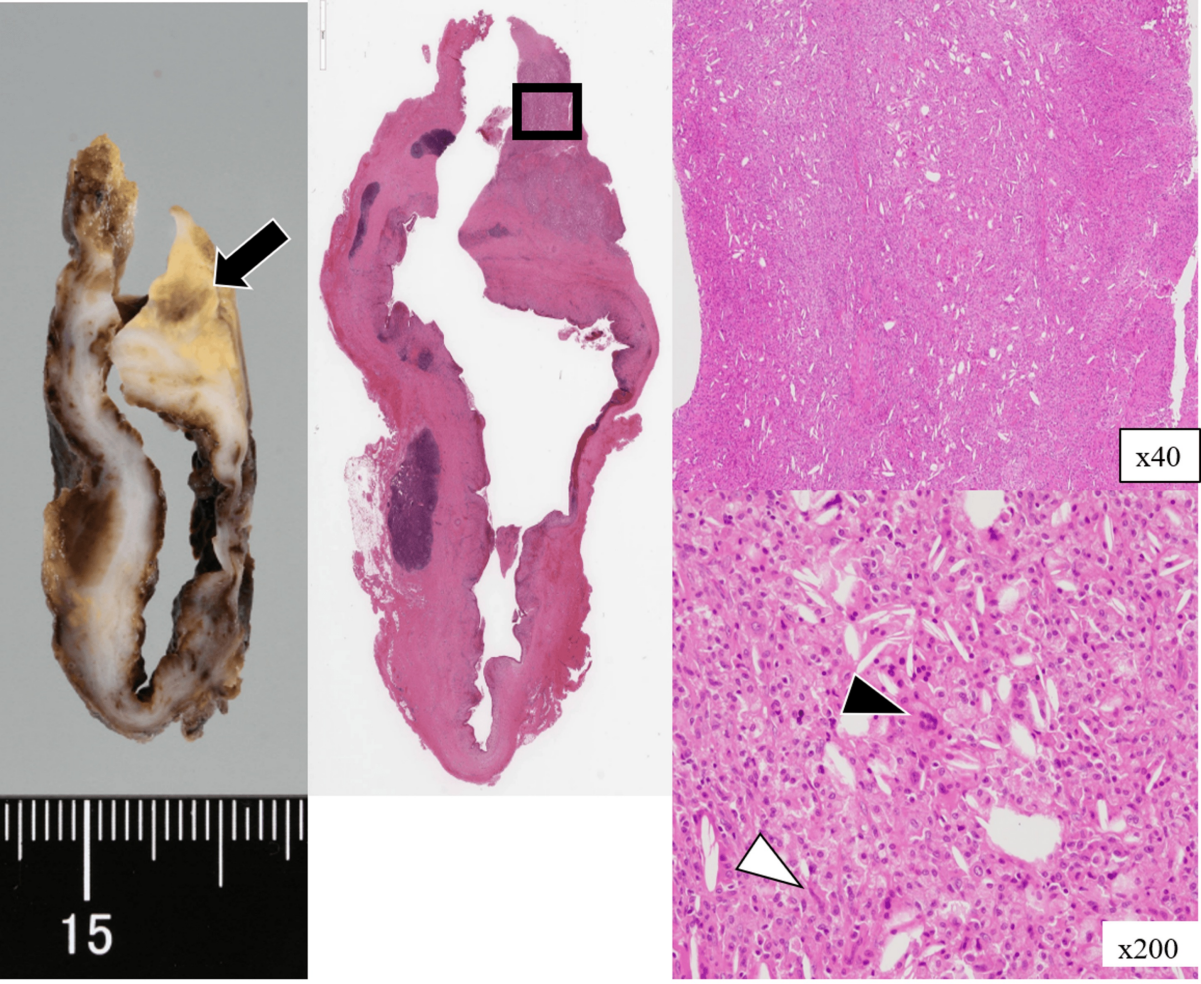
Histopathological findings Gross specimen (left): thick-walled cyst with focal yellow white plaques (black arrow). Histology (H&E) (middle and right): foamy macrophages (white arrowhead) and Touton giant cells (black arrowhead) beneath stratified‑squamous lining (×200).

The 36 × 33 × 26 mm specimen contained turbid fluid and focal yellow‑white plaques (Figure 3: left). Histology confirmed a stratified-squamous-lined cyst with dense xanthogranulomatous infiltrate of foamy macrophages, Touton giant cells, and cholesterol clefts (Figure 3: middle and right).

The postoperative course was uneventful, and the patient remains recurrence-free 3 months after surgery.

## Discussion

XGI complicating BCCs is extremely rare. A systematic search revealed only two adult cases reported by Sarioglu et al. [8] and Chaturvedi and Chaturvedi [9], to which the present case adds a third (Table 1). 

**Table 1:** Comparison of adult branchial cleft cysts with xanthogranulomatous inflammation SCM: sternocleidomastoid, IJV: internal jugular vein

Author, year	Age/sex	Size (cm)	Adhesion site	Frozen section
Sarioglu et al. (2012) [8]	39 F	3.5	SCM fascia	XGI
Chaturvedi and Chaturvedi (2022) [9]	23 M	2.0	Digastric muscle, IJV	XGI
Present case 2025	71 M	3.3	Carotid sheath, IJV	XGI

All three patients presented with painless, slowly enlarging lateral‑neck masses whose ultrasonographic or cross-sectional imaging appearances were indistinguishable from uncomplicated BCCs; however, each lesion displayed disproportionate fibrosis and firm adhesions that intra‑operatively mimicked invasive malignancy. In our case, the cyst was tightly attached to the carotid sheath, whereas adhesions involved the sternocleidomastoid fascia in Sarioglu et al.’s report and the digastric muscle/internal jugular vein in Chaturvedi and Chaturvedi’s report. These findings emphasize that routine imaging cannot reliably distinguish XGI-associated cysts from metastatic or infected counterparts.

Current evidence suggests that chronic leakage or rupture of cyst contents triggers a macrophage-mediated xanthogranulomatous response via exposure to keratin and cholesterol crystals [8, 9]. Our specimen exhibited abundant cholesterol clefts and dense wall fibrosis, consistent with long-standing inflammatory stimulation. FNA may potentiate this reaction. It may be advisable to avoid performing cytological aspiration with excessive force or an aggressive technique. FNA preceded surgery in both our patient and Chaturvedi and Chaturvedi’s case, while XGI has also arisen in a thyroglossal‑duct cyst after FNA [10]. Although causality remains unproven, surgeons should recognize that antecedent aspiration can foster florid histiocytic inflammation that masquerades as tumor infiltration. The intraoperative frozen section was pivotal in all published cases. Rapid demonstration of foamy macrophages and Touton giant cells, without epithelial atypia, excluded malignancy and permitted conservative excision without radical neck dissection. Therefore, these findings indicate that implementing intraoperative rapid diagnostic assessment - particularly frozen-section analysis - is highly advantageous in BCC cases that exhibit adhesions. Follow-up ranging from 6 to 12 months confirmed uneventful healing and absence of recurrence, indicating that wide oncologic margins are unnecessary once benign histology is established. Thus, the combination of benign‑appearing cystic imaging, unexpectedly tenacious adhesions, and frozen‑section evidence of XGI should alert clinicians to this rare entity. Comprehensive histopathological sampling remains essential to rule out an occult carcinoma component, as XGI has occasionally co-existed with malignancy at other sites.

## Conclusions

XGI superimposed on a BCC is an uncommon malignant mimicker. In our patient, pronounced adhesions were present; nevertheless, the lesion was sharply dissected and removed en bloc without any intraoperative or postoperative complications. Specifically, when a BCC is diagnosed pre-operatively but unexpectedly dense adhesions are encountered intraoperatively, XGI should be included in the differential; an intraoperative frozen-section examination ought to be performed to secure an accurate diagnosis and to guide the extent of resection.
